# The association between serum anion gap and acute kidney injury after coronary artery bypass grafting in patients with acute coronary syndrome

**DOI:** 10.1186/s12872-023-03588-y

**Published:** 2023-11-08

**Authors:** Qinyuan Pan, Zhifang Mu, Yong Li, Caihong Gu, Tao Liu, Bing Wang, Xiuwen Kang

**Affiliations:** 1 Department of Critical Care Medicine, The First People’s Hospital of Lianyungang, Zhenhua East Road, Lianyungang, 222002 Jiangsu China; 2 Department of Cardiology, Jinshan Branch of Shanghai Sixth People’s Hospital, Shanghai, 201500 China

**Keywords:** Acute kidney injury, Acute coronary syndrome, Coronary artery bypass grafting, Intensive care unit, Serum anion gap

## Abstract

**Background:**

The purpose of this study was to explore the association between serum anion gap (SAG) and acute kidney injury (AKI) after coronary artery bypass grafting (CABG) in patients with acute coronary syndrome (ACS) in the Intensive Care Unit (ICU).

**Methods:**

We retrospectively analyzed the clinical data of 2,428 ACS patients who underwent CABG in the Medical Information Mart for Intensive Care IV (Mimic-IV) database. The endpoint of this study was AKI after CABG. The baseline data of the two groups (non-AKI group vs. AKI group) was compared, and the restricted cubic spline (RCS) plot, multivariable logistic regression model, and subgroup analysis were used to explore the relationship between SAG and the risk of AKI after CABG.

**Results:**

In the adjusted multivariate logistic regression model, SAG was an independent predictor of AKI after CABG (OR = 1.12, 95% CI: 1.02–1.23, *P* = 0.015). The RCS revealed that the relationship between SAG levels and risk of AKI was J-shaped. When the SAG was ≥ 11.58 mmol/L, the risk of AKI increased by 26% for each unit increase in SAG. Additionally, we further divided the SAG into quartiles. In the fully adjusted model, compared with the first quartile of SAG, the odds ratios (ORs) and 95% confidence intervals (CIs) for AKI risk across the SAG quartiles were 0.729 (0.311, 1.600), 1.308 (0.688–2.478), and 2.221 (1.072, 4.576).

**Conclusions:**

The SAG level was associated with the risk of AKI after CABG in a J-shaped curve in the ICU. However, the underlying causes of the problem need to be investigated.

**Supplementary Information:**

The online version contains supplementary material available at 10.1186/s12872-023-03588-y.

## Introduction

Acute coronary syndrome (ACS) is a group of clinical syndromes characterized by atherosclerotic plaque rupture, hemorrhage and thrombosis with high morbidity, rapid changes in disease, and a critical prognosis, making it one of the deadliest diseases worldwide [1, 2]. Currently, coronary artery bypass grafting (CABG) is a cardiac surgical procedure to improve myocardial blood supply by repairing or replacing an obstructed coronary artery, and it is an effective treatment for ACS [3, 4]. Patients with CABG have poor cardiac function themselves, as well as the occurrence of myocardial impairment, vasodilatory malfunction, electrolyte acid-base disturbance and cardiac compression, which induce the development of hypovolemic syndrome after surgery [5, 6]. The kidney is a more sensitive organ to low perfusion, and a more common and serious complication in these patients is acute kidney injury (AKI) when cardiac output is reduced. Hayıroğlu M et al. demonstrated that in patients with ST-segment elevation myocardial infarction (STEMI) complicated by cardiogenic shock and treated with primary percutaneous coronary intervention (pPCI), AKI was an independent prognostic factor for long-term mortality [7]. The kidney functions play a major role in risk scores such as the intermountain risk score (IMRS). Çınar T et al. found that the patients with STEMI can be predicted both short- and long-term based on IMRS. Additionally, the predictive value of the IMRS for overall mortality was non-inferior to that of the TIMI and GRACE scores [8]. When AKI occurs after CABG in patients with ACS, it can increase postoperative mortality [9, 10]. Thus, obtaining valid indicators to observe the presence of AKI after CABG is an important measure to reduce mortality in these patients. Several parameters can also predict AKI injury in the follow-up of coronary artery disease. An increase in platelet-to-lymphocyte levels in patients undergoing primary percutaneous coronary intervention for STEMI is independently associated with a greater risk of CI-AKI [11].

The equation, SAG = [Na^+^ (mmol/L) + K^+^ (mmol/L)] − [Cl^−^ (mmol/L) + HCO3^−^ (mmol/L)], was widely used [12]. In the ICU, serum anion gap (SAG) is one of the simple, easily available, rapid and effective biomarkers [13, 14]. The SAG levels have been studied in relation to the prognosis of critically ill patients [15]. Chen Q et al. found that the ICU mortality in patients with aortic aneurysms may be predicted by the SAG at admission [16]. Sahu A and his team also revealed that in acute myocardial infarction, the initial SAG is a predictor of mortality [17]. In addition, Yildiz I et al. revealed that the utilization of serum osmolarity, as a result of its straightforward calculation upon admission, may serve as a valuable tool in identifying patients with STEMI undergoing pPCI who are more likely to develop contrast-induced nephropathy (CIN) [18]. The amalgamation of clinical and anatomic factors can enhance the precision of identifying patients who are at high risk for CIN after pPCI. Contrary to isolated Syntax Score, higher Score II has been found to indicate an increase in CIN risk in STEMI patients undergoing pPCI [19]. However, the relationship between SAG levels and the development of acute kidney injury (AKI) in ACS patients after CABG is unclear. Medical Information Mart for Intensive Care IV (MIMIC-IV) is a large, single-center U.S. intensive care medical information database that contains information on all critical care patients admitted to the intensive care unit from 2008 to 2019 [20]. Therefore, we investigated the relationship between SAG levels and the risk of AKI by retrieving and analyzing the clinical data of ACS patients after CABG in the MIMIC-IV database to provide a scientific reference basis for early prognosis and effective treatment planning in clinical practice.

## Materials and methods

### Study population

**Fig. 1:**
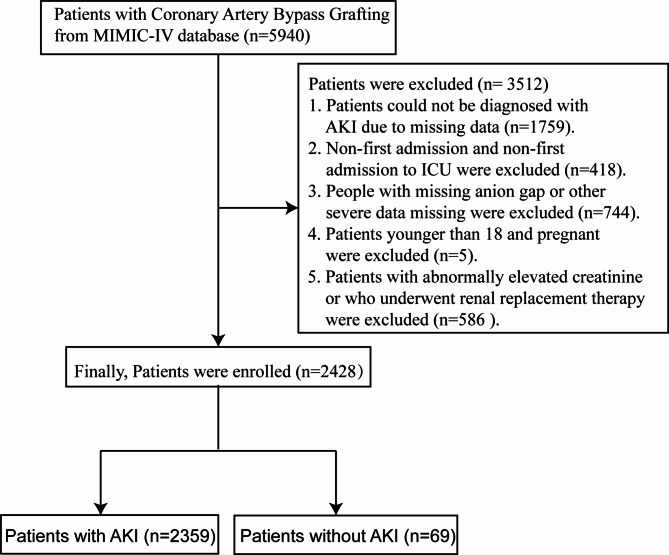
Flowchart of patient selection. Abbreviation: AKI, acute kidney injury; ICU, Intensive Care Unit; Mimic-IV, Medical Information Mart for Intensive Care IV.

A total of 5,940 ACS patients who underwent CABG in the MIMIC-IV database were retrospectively obtained (ICD 9-code: 361, 021). Exclusion criteria were as follows: (1) patients who could not conclude whether they had AKI due to missing creatinine measurements; (2) patients who were not tested for SAG at the time of ICU admission or who had severe missing baseline data; (3) Non-first admission and non-first admission to ICU were excluded; and (4) Patients younger than 18 and pregnant were excluded; (5) Patients with abnormally elevated creatinine or who underwent renal replacement therapy were excluded. Finally, 2428 patients were included (Fig. 1). Any researcher who adheres to the data use requirements is permitted access to the database, and our certificate number is 35,942,590. The study complied with medical ethics requirements.

### Variables

Baseline information about the patients was recorded, including demographics (age, gender, race, BMI, systolic blood pressure (SBP), diastolic blood pressure (DBP), laboratory tests (albumin, lactic acid, white blood cells (WBC), lymphocytes, neutrophils, bicarbonate, blood urea nitrogen (BUN), serum calcium, serum chloride, serum creatinine (Scr), glucose, serum sodium, serum potassium, hematocrit, hemoglobin (Hb), platelets, red blood cell (RBC), red blood cell distribution width (RDW), C-reactive protein (CRP), and medication history (cardiotonic, diuretics, vasoactive drugs). The following complications also were included: hypertension, diabetes mellitus (DM), congestive heart failure (CHF), atrial fibrillation (AF), acute myocardial infarction (AMI), respiratory failure (RF), chronic obstructive pulmonary disease (COPD), ventricular fibrillation (VF), and cardiogenic shock. All baseline data were used from the first ICU admission for the first time, and the Sofa score was the first score after admission to the ICU. In addition, for all patients admitted to the ICU more than once, clinical data also from the first ICU admission were selected.

### Subgroups and endpoint events

Patients admitted to the ICU after CABG were divided into the AKI group (69 patients) and the non-AKI group (2,359 patients) based on whether they had AKI. The endpoint event in this study was the occurrence of AKI, which was defined as an increase in Scr level ≥ 0.3 mg/dl (≥ 26.5 mmol/L) or more than 1.5 times the basal value within 7 days, or urine volume ≤ 0.5 ml/kg/h for 6 h, according to the Kidney Disease: Improving Global Outcomes diagnostic criteria for AKI [21].

### Statistical analysis

Normally distributed data were expressed as mean ± standard deviation, and the t-test or Wilcoxon rank sum test was used for comparison between groups. Count data were expressed as percent composition ratio (%), and the chi-square test was used for comparison between groups. Data with statistically significant differences were screened out after univariate logistic regression analysis, followed by multivariate logistic regression analysis. The restricted cubic spline (RCS) model and multivariate logistic regression analysis were used to analyze the relationship between SAG levels and AKI, and finally, subgroup analysis was performed to analyze whether there was an interaction between SAG and age, gender, BMI, and other concomitant diseases (hypertension, and DM). Stata 15.0 and RStudio 4.1.1 software were used for data analysis. A *P*-value < 0.05 was considered statistically significant difference.

## Results

### Baseline characteristics

As shown in Table 1, compared to the non-AKI group, patients in the AKI group had a higher BMI and a higher probability of combined CHF, AF, RF, VF, and cardiogenic shock, but a lower probability of combined hypertension. In addition, albumin and serum calcium were lower, WBC, lymphocytes, neutrophils, BUN, Scr, RDW, CRP, Sofa score, SAG, and corrected SAG were higher, and cardiotonic and vasoactive drugs were more likely to be used (all *P* < 0.05), while other baseline information was not statistically significant between the two groups (all *P* > 0.05).

**Table 1:** Baseline Characteristics of the Non-AKI and AKI patients

Variables	Overall (n = 2,428)	Non-AKI group (n = 2,359)	AKI group (n = 69)	*P*-value
Age, years	66.95 ± 10.25	66.92 ± 10.22	67.91 ± 11.20	0.427
Gender, (%)				0.209
Male	2338 (96.3%)	2274 (93.7%)	64 (2.6%)	
Female	90 (3.7%)	85 (3.5%)	5 (0.2%)	
Race, (%)				0.940
White	1685 (69.5%)	1637 (69.4%)	48 (69.6%)	
Black	56 (2.2%)	54 (2.3%)	2 (2.9%)	
Other	687 (28.3%)	668 (28.3%)	19 (27.5%)	
BMI, kg/m^2^	29.03 ± 5.23	28.99 ± 5.22	30.52 ± 5.38	0.017
SBP, mmHg	112.64 ± 15.37	112.69 ± 15.40	111.04 ± 14.28	0.380
DBP, mmHg	59.05 ± 9.90	59.10 ± 9.88	57.36 ± 10.59	0.151
CHF, (%)	198 (8.2%)	186 (7.9%)	12 (17.4%)	0.009
AF, (%)	910 (37.5%)	873 (37.0%)	37 (53.6%)	0.007
AMI, (%)	381 (15.7%)	364 (15.4%)	17 (24.6%)	0.057
RF, (%)	134 (5.5%)	118 (5.0%)	16 (23.2%)	< 0.001
COPD, (%)	84 (3.5%)	83 (3.5%)	1 (1.4%)	0.553
VF, (%)	36 (1.5%)	26 (1.1%)	10 (14.5%)	< 0.001
Hypertension, (%)	796 (32.8%)	782 (33.1%)	14 (20.3%)	0.035
DM, (%)	437 (18.0%)	425 (18.0%)	12 (17.4%)	0.894
Cardiogenic shock, (%)	99 (4.1%)	88 (3.7%)	11 (15.9%)	< 0.001
SAG, mmol/L	12.78 ± 2.89	12.75 ± 2.86	13.81 ± 3.73	0.003
Corrected SAG, mmol/L	14.06 ± 3.22	14.02 ± 3.18	15.61 ± 4.07	0.002
Bicarbonate, mmol/L	25.87 ± 3.22	25.88 ± 3.21	25.43 ± 3.52	0.253
BUN, mg/dl	18.60 ± 6.59	18.53 ± 6.54	21.01 ± 7.92	0.002
Calcium, mg/dl	8.61 ± 0.52	8.62 ± 0.52	8.46 ± 0.57	0.013
Chloride, mmol/L	102.29 ± 4.58	102.29 ± 4.57	102.04 ± 4.74	0.653
Scr, mg/dL	0.94 ± 0.25	0.94 ± 0.24	1.05 ± 0.31	< 0.001
Glucose, mg/dL	123.97 ± 29.59	123.76 ± 29.47	131.21 ± 32.80	0.039
Sodium, mmol/L	137.89 ± 3.20	137.90 ± 3.21	137.62 ± 3.01	0.473
Potassium, mmol/L	4.20 ± 0.39	4.20 ± 0.39	4.18 ± 0.45	0.644
Hematocrit, %	32.08 ± 5.75	32.11 ± 5.77	31.21 ± 5.00	0.200
Hb, g/dl	10.76 ± 2.00	10.77 ± 2.00	10.51 ± 1.82	0.280
Platelet, ×10^9^/L	185.75 ± 66.63	185.59 ± 66.53	191.10 ± 70.18	0.498
RBC, ×10^9^/L	3.56 ± 0.67	3.56 ± 0.67	3.43 ± 0.57	0.109
RDW, %	13.76 ± 1.11	13.74 ± 1.11	14.18 ± 1.25	0.001
CRP, mg/L	42.66 ± 33.38	42.20 ± 33.14	58.59 ± 37.81	< 0.001
Albumin, g/dL	3.99 ± 0.48	3.99 ± 0.48	3.78 ± 0.52	< 0.001
Lactic acid, mmol/L	2.10 ± 0.88	2.10 ± 0.88	2.03 ± 1.00	0.513
WBC, ×10^9^/L	9.10 ± 3.41	9.04 ± 3.38	11.43 ± 3.88	< 0.001
Lymphocytes, ×10^9^/L	1.96 ± 0.73	1.95 ± 0.72	2.35 ± 0.80	< 0.001
Neutrophils, ×10^9^/L	6.33 ± 2.86	6.28 ± 2.83	8.14 ± 3.34	< 0.001
Cardiotonic, (%)	195 (8.0%)	179 (7.6%)	16 (23.2%)	< 0.001
Vasoactive drugs, (%)	2410 (99.3%)	2342 (99.3%)	68 (98.6%)	0.406
Diuretics, (%)	118 (4.9%)	109 (4.6%)	9 (13.0%)	0.003
SOFA score	2.72 ± 2.12	2.67 ± 2.09	4.21 ± 2.44	< 0.001

Abbreviations: AKI, acute kidney injury; AHF, Acute Heart failure; BMI, body mass index; SBP, systolic blood pressure; DBP, diastolic blood pressure; AF, atrial fibrillation; AMI, acute myocardial infarction; RF, respiratory failure; VF, ventricular fibrillation; SAG, serum anion gap; BUN, blood urea nitrogen; Scr, serum creatinine; Hb, hemoglobin; RBC, red blood cell; RDW, red cell distribution width; CRP, C-reactive protein; WBC, white blood cell; SOFA, sequential organ failure assessment

### Association between SAG and AKI

**Fig. 2:**
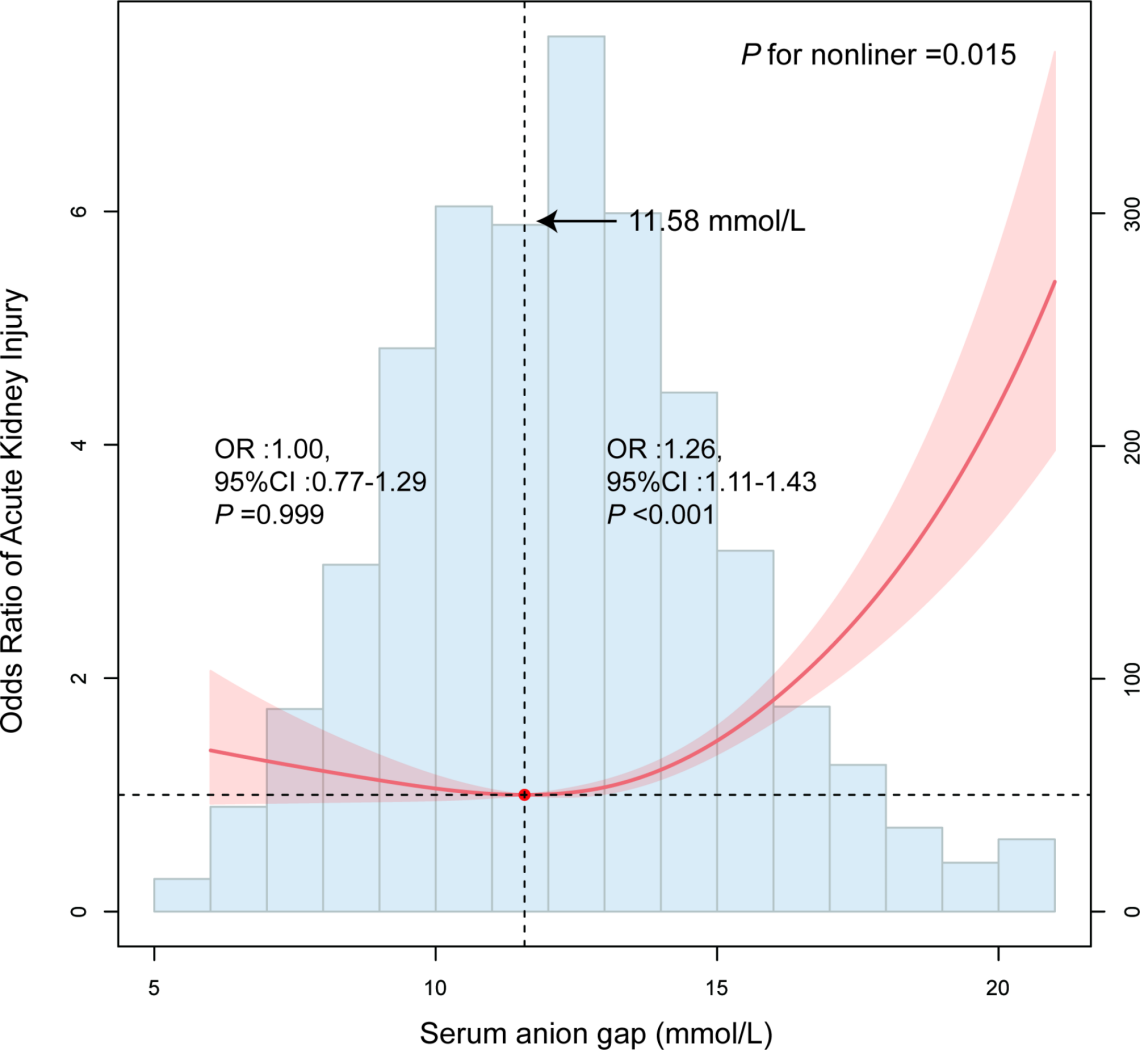
Restricted cubic spline plot of the association between SAG and the risk of AKI after CABG. Abbreviation: SAG, serum anion gap; AKI, acute kidney injury; CABG, coronary artery bypass grafting

Univariate logistic regression analysis showed that serum anion gap was a predictor of AKI (OR: 1.13, 95% CI: 1.04–1.22, *P* = 0.003). Then, according to univariate analysis, statistically significant indicators (*P* < 0.05) were included in multivariate logistic regression analysis, including SAG, BMI, VF, hypertension, WBC, serum calcium, furosemide, and Sofa score. SAG was still an independent predictor of AKI after CABG (OR = 1.12, 95% CI: 1.02–1.23, *P* = 0.015, Table 2). The correlation between SAG and AKI was analyzed by the RCS plot, as shown in Fig. 2, SAG showed a J-curve relationship with AKI (*P* = 0.015), and the cut-off value of SAG was 11.58 mmol/L. Further analysis of the relationship between SAG at both sides of 11.58 mmol/L and AKI showed that when SAG ≥ 11.58 mmol/L, the risk of AKI increased by 26% for each unit increase in SAG. We divided the SAG concentration into quartiles (Q1: 5.00–11.00 mmol/L; Q2: 11.01-13.00 mmol/L; Q3: 13.01-15.00 mmol/L; Q4: 15.01-21.00 mmol/L). After adjusting for confounding factors, including age, gender, race, hypertension, DM, BMI, CHF, AF, RF, VF, cardiogenic shock, albumin, WBC, BUN, serum calcium, Scr, glucose, RDW, CRP, cardiotonic, diuretic, and Sofa score, compared with the lowest quartiles, the odds ratios (ORs) with 95% confidence intervals (CIs) for AKI across the quartiles were 0.729 (0.311, 1.600), 1.308 (0.688–2.478), and 2.221 (1.072, 4.576) for SAG (Table 3). Additionally, we also found that the corrected SAG was associated with risk of AKI in the J-shaped relationship (Supplementary Tables 1, and Supplementary Fig. 1).

**Table 2:** Univariate and multivariate logistic regression of SAG in predicting AKI after CABG

Variables	Univariate logistic regression			Multivariate logistic regression	
	OR (95% CI)	*P*-Value		OR (95% CI)	*P*-Value
SAG	1.13 (1.04–1.22)	0.003		1.12 (1.02–1.23)	0.015
BMI	1.05 (1.01–1.10)	0.017		1.05 (1.00-1.09)	0.043
CHF	2.46 (1.3–4.67)	0.006		0.67 (0.28–1.48)	0.338
AF	1.97 (1.22–3.18)	0.006		1.49 (0.88–2.51)	0.133
RF	5.73 (3.18–10.32)	< 0.001		2.11 (0.95–4.50)	0.059
VF	15.21 (7.01–32.99)	< 0.001		8.20 (3.17–20.44)	< 0.001
Hypertension	0.51 (0.28–0.93)	0.027		0.54 (0.28–0.98)	0.049
Cardiogenic shock	4.89 (2.48–9.64)	< 0.001		1.40 (0.49–3.70)	0.514
Albumin	0.43 (0.27–0.68)	< 0.001		0.76 (0.44–1.33)	0.337
WBC	1.19 (1.12–1.26)	< 0.001		1.13 (1.05–1.21)	0.001
Lymphocytes	1.76 (1.37–2.26)	< 0.001		0.95 (0.36–2.67)	0.914
Neutrophils	1.20 (1.12–1.29)	< 0.001		0.76 (0.32–1.86)	0.539
BUN	1.05 (1.02–1.09)	0.002		0.98 (0.94–1.03)	0.410
Serum calcium	0.55 (0.34–0.88)	0.013		0.41 (0.23–0.69)	0.001
Scr	5.35 (2.21–12.94)	< 0.001		2.39 (0.76–7.65)	0.140
Glucose	1.01 (1.00-1.02)	0.040		1.00 (0.99–1.01)	0.868
RDW	1.37 (1.13–1.66)	0.001		0.99 (0.78–1.25)	0.937
CRP	1.01 (1.01–1.02)	< 0.001		1.00 (0.99-1.00)	0.380
Cardiotonic	3.68 (2.06–6.57)	< 0.001		1.63 (0.74–3.42)	0.212
Furosemide	3.10 (1.50–6.41)	0.002		0.22 (0.07–0.67)	0.009
Sofa score	1.34 (1.21–1.48)	< 0.001		1.26 (1.13–1.41)	< 0.001

Abbreviations: SAG, serum anion gap; AKI, acute kidney injury; CABG, coronary artery bypass grafting; CHF, congestive heart failure; BMI, body mass index; AF, atrial fibrillation; RF, respiratory failure; VF, ventricular fibrillation; WBC, white blood cell; BUN, blood urea nitrogen; Scr, serum creatinine; RDW, red cell distribution width; CRP, C-reactive protein; Sofa, sequential organ failure assessment

**Table 3:** Association between SAG and the risk of AKI after CABG

SAG	Model 1	Model 2	Model 3
Q1, mmol/L	Ref.	Ref.	Ref.
Q2, mmol/L	0.718 (0.308, 1.545)	0.779 (0.330, 1.710)	0.729 (0.311, 1.600)
Q3, mmol/L	1.195 (0.628, 2.266)	1.283 (0.664, 2.478)	1.308 (0.688–2.478)
Q4, mmol/L	2.308 (1.232, 4.339) **	2.399 (1.271, 4.549) **	2.221 (1.072, 4.576) *
*P* for trend	0.049	0.033	0.132

Abbreviation: SAG, serum anion gap; AKI, acute kidney injury; CABG, coronary artery bypass grafting; Q1, 5.00–11.00 mmol/L; Q2, 11.01-13.00 mmol/L; Q3, 13.01-15.00 mmol/L; Q4, 15.01-21.00 mmol/L; **P < 0.01, *P < 0.05; Model 1 was adjusted for age, and gender. Model 2 was adjusted for age, gender, race, hypertension, diabetes mellitus, and body mass index. Model 3 was adjusted for age, gender, race, hypertension, diabetes mellitus, body mass index, congestive heart failure, atrial fibrillation, respiratory failure, ventricular fibrillation, cardiogenic shock, albumin, white blood cell, lymphocytes, neutrophils, blood urea nitrogen, calcium, serum creatinine, glucose, red cell distribution width, C-reactive protein, cardiotonic, furosemide, and sequential organ failure assessment

### Subgroup analyses

As shown in Table 4, in the subgroup analysis, we included age, gender, hypertension, DM, and BMI. SAG and AKI had a significant J-shaped relationship among participants with DM. (*P* for trend = 0.023). It was found that no significant interaction was observed except for DM (*P* for interaction = 0.007). Therefore, there was an interaction between SAG and the development of AKI in patients with CABG with DM but no significant interaction with other risk factors.

**Table 4:** Subgroup analysis for associations between SAG and the risk of AKI after CABG

SAG	Q1	Q2	Q3	Q4	*P* for trend	*P* for interaction
	OR (95%CI)	OR (95%CI)	OR (95%CI)	OR (95%CI)		
Age						0.256
< 60	1.00	0.444 (0.036, 3.759)	1.202 (0.610, 2.937)	2.126 (0.412, 4.894)	0.287	
≥ 60	1.00	0.663 (0.222, 1.983)	1.237 (0.547, 2.798)	2.786 (1.132, 6.859) *	0.086	
Gender						0.345
Male	1.00	0.826 (0.392,1.737)	0.652 (0.271, 2.569)	2.029 (0.926, 4.448)	0.178	
Female	1.00	0.710 (0.348, 1.447)	0.583 (0.257, 1.323)	1.662 (0.847, 3.263)	0.274	
Hypertension						0.516
No	1.00	0.992 (0.372, 2.642)	1.458 (0.666, 3.192)	2.836 (1.129, 7.127) *	0.077	
Yes	1.00	0.475 (0.046, 4.876)	1.020 (0.201, 5.192)	1.188 (0.172, 8.218)	0.950	
DM						0.007
No	1.00	0.797(0.304, 2.089)	1.456 (0.701, 3.025)	1.766 (0.694, 4.495)	0.476	
Yes	1.00	0.456 (0.017, 1.241)	1.491 (0.096, 2.311)	5.834 (1.363, 6.840) *	0.023	
BMI						0.496
< 30 kg/m^2^	1.00	0.798 (0.250, 2.550)	1.292 (0.511, 3.269)	1.563(0.501, 4.878)	0.664	
≥ 30 kg/m^2^	1.00	0.710 (0.158, 3.196)	1.555 (0.515, 4.697)	3.901 (1.141, 1.333) *	0.086	

Abbreviations: SAG, serum anion gap; AKI, acute kidney injury; CABG, coronary artery bypass grafting; Q1, 5.00–11.00 mmol/L; Q2, 11.01-13.00 mmol/L; Q3, 13.01-15.00 mmol/L; Q4, 15.01-21.00 mmol/L; *P < 0.05; OR, odd ratio; CI, confidence interval. Analysis was adjusted for age, gender, race, hypertension, diabetes mellitus, body mass index, congestive heart failure, atrial fibrillation, respiratory failure, ventricular fibrillation, cardiogenic shock, albumin, white blood cell, lymphocytes, neutrophils, blood urea nitrogen, calcium, serum creatinine, glucose, red cell distribution width, C-reactive protein, cardiotonic, furosemide, and sequential organ failure assessment

## Discussion

Critically ill patients often have a combination of acid-base balance disorders, especially metabolic acidosis, which often increases the risk of developing AKI in patients [22]. Clinicians have been using SAG as the main tool to assess the state of acid-base disorders, and it is valuable in the differential diagnosis of the etiology and type of metabolic acidosis [14, 23]. When metabolic acidosis occurs, SAG levels are elevated [24]. AKI is a clinical complication in patients after coronary artery bypass grafting primarily due to a sudden decline in renal function caused by low cardiac output syndrome, which can seriously affect patient prognosis and significantly increase the risk of postoperative death [25].

In the present study, firstly, the SAG levels were found to be significantly higher in the AKI group compared to the N-AKI group (*P* = 0.003) by the population baseline data sheet. Secondly, by univariate and multifactorial logistic analysis, we found that changes in SAG levels were an independent risk factor for the development of AKI in patients after coronary artery bypass grafting. According to the restricted cubic spline shown, we could observe that SAG level showed a J-shaped curve relationship with the occurrence of AKI. The risk of AKI in patients decreased with increasing SAG levels and increased when the inflection point of 11.58 mmol/L was reached. Finally, in a subgroup analysis stratified by age, gender, hypertension, DM, and BMI based on the results of the interaction test (*P* for interaction = 0.007), we concluded that differences in SAG levels have different effects on AKI in patients after CABG with or without a history of DM.

Risk and outcome differ between different types of cardiac surgery (aorta, valve, CABG). Chen JJ et al. found that when compared with other kinds of heart surgeries, aortic surgery caused a greater percentage of dialysis requiring AKI [26]. In addition, Li Q and his team also revealed that the level of NT-proBNP before cardiac surgery has been confirmed to be strongly related to the risk of AKI following cardiac surgery [27]. Previous studies have shown that SAG levels are associated with the prognosis of cardiovascular disease [28, 29]. In the coronary care unit, the risk of AKI and in-hospital all-cause mortality increased with increasing SAG quartile levels. The Chenbo Xu research team found that higher SAG levels were significantly associated with increased all-cause mortality at 30 days, 180 days, and 1 year in patients with acute myocardial infarction [30]. Charles I McDonald et al. revealed that SAG levels were significantly associated with increased risk of death in patients with cardiogenic shock. SAG levels were significantly higher in the non-surviving group compared to the surviving group, and the best cut-off value for predicting mortality was a SAG greater than 6 mmol/L during the first 24 h of extracorporeal membrane pulmonary oxygenation [31]. Yiyang Tang et al. showed a U-shaped relationship between SAG levels and 30-day all-cause mortality in critically ill patients with congestive heart failure [32]. In addition, Bihuan Cheng et al. also found the same U-shaped relationship between SAG levels and 30-day all-cause mortality in critically ill patients in the ICU [33]. In the diabetic population, altered SAG levels are significant for the development of AKI. However, in the non-diabetic population, changes in SAG levels were not meaningful for the occurrence of AKI. This may be due to abnormal SAG that can be caused in diabetic patients due to disorders of glucose metabolism and hormone secretion. In addition, Yingchao Zhang’s team found that higher SAG was associated with the risk of impaired fasting glucose, or DM [34]. The body regulates and maintains acid-base balance through three pathways: the humoral buffer system, the lungs and the kidneys, and the compensation of the kidney pathway takes several days. DM leads to impaired renal function, weakened acid-base balance of kidney, and unable to produce more bicarbonate to compensate in a short period of time, so DM group has higher SAG levels. Patients with diabetes usually have higher blood sugar than patients without diabetes, which makes patients with diabetes more likely to develop acute kidney injury [35]. In addition, patients with diabetes are more susceptible to high SAG metabolic acidosis than patients without diabetes [36]. Thus, the association of higher SAG with outcomes might be influenced by DM. Therefore, controlling SAG at relatively low levels may help prevent impaired fasting glucose, or DM. The SAG can also be affected by the lactate level in the blood. Lactate is an important parameter that can affect in-hospital endpoints. Based on univariable and multivariable Cox proportional survival analyses, Mert İlker H et al. found that lactate levels can be used to predict short- and long-term mortality in patients with cardiogenic shock [37]. Hayıroğlu M and his team revealed that lactic acid level is an independent predictor of in-hospital death in patients with STEMI complicated by cardiogenic shock [38].

The SAG is used to evaluate electrolytes and acid-base balance. A series of studies have shown that SAG plays an important role in the diagnosis and prognosis of a variety of acute and chronic diseases. The present study concluded that SAG showed a J-shaped relationship with the occurrence of AKI based on the MIMIC IV database. At the same time, this study has some limitations. First, this study is a retrospective study, which may introduce selection bias. Second, we observed the endpoint event as the occurrence of AKI after CABG in ACS patients in the ICU and did not further observe the prognosis of patients after CABG when AKI occurred. Third, our findings were derived from the single-center Mimic-IV database, and further external validation is needed.

## Conclusion

In conclusion, the SAG level was associated with the risk of AKI after CABG in a J-shaped curve in the ICU. The risk of AKI after CABG and SAG level were positively correlated when the serum AG value was > 11.58 mmol/L. In addition, differences in SAG levels have different effects on AKI in patients after CABG with or without a history of DM. Therefore, SAG might be served as an indicator of early risk stratification.

### Electronic supplementary material

Below is the link to the electronic supplementary material.

Supplementary Material 1

Supplementary Material 2

## Data Availability

This study’s clinical information was extracted from MIMIC-IV database (https://mimic.physionet.org/). Before requesting permission to use this open and free database, researchers must take the Protecting Human Research Participants online course at the National Institutes of Health.
