# Supplementary material for: The association between serum anion gap and acute kidney injury after coronary artery bypass grafting in patients with acute coronary syndrome
Source: BMC Cardiovasc Disord. 2023 Nov 8;23:542. doi: 10.1186/s12872-023-03588-y (PMC10634147; doi:10.1186/s12872-023-03588-y)
Supplement: Supplementary file 1 — Supplementary Material 1 [file 12872_2023_3588_MOESM1_ESM.docx]

| **Supplementary Table 1.** Association between corrected SAG and the risk of AKI after CABG   \| Variable \| OR (95% CI) \| \| \| \| --- \| --- \| --- \| --- \| \| Corrected SAG \| 1.118 (1.022-1.224) * \| \| \| \| Interquartile \|  \|  \|  \| \| Q1 \| Ref. \| \| \| \| Q2 \| 0.699 (0.289-1.596) \| \| \| \| Q3 \| 1.183 (0.532-2.614) \| \| \| \| Q4 \| 1.529 (0.670-3.515) \| \| \| \| *P* for trend \| 0.190 \| \| \| |
| --- | --- | --- | --- | --- | --- | --- | --- | --- | --- | --- | --- | --- | --- | --- | --- | --- | --- | --- | --- | --- | --- | --- | --- | --- | --- | --- | --- | --- | --- | --- | --- | --- |

Abbreviation: SAG, serum anion gap; AKI, acute kidney injury; CABG, coronary artery bypass grafting; Q1, 4.25-12.00 mmol/L; Q2, 12.01-14.00 mmol/L; Q3, 14.01-16.00 mmol/L; Q4, 16.00-25.25 mmol/L; OR, odds ratio; CI, confidence interval; Model 1 was adjusted for age, and gender. Model 2 was adjusted for age, gender, race, hypertension, diabetes mellitus, and body mass index. Model 3 was adjusted for age, gender, race, hypertension, diabetes mellitus, and body mass index, congestive heart failure, [atrial fibrillation](javascript:;), respiratory failure, ventricular fibrillation, cardiogenic shock, albumin, white blood cell, lymphocytes, neutrophils, blood urea nitrogen, calcium, serum creatinine, glucose, red cell distribution width, C-reactive protein, cardiotonic, furosemide, sequential organ failure assessment.
