# Supplementary material for: The association between serum anion gap and acute kidney injury after coronary artery bypass grafting in patients with acute coronary syndrome
Source: BMC Cardiovasc Disord. 2023 Nov 8;23:542. doi: 10.1186/s12872-023-03588-y (PMC10634147; doi:10.1186/s12872-023-03588-y)
Supplement: Supplementary file 2 — Supplementary Material 2 [file 12872_2023_3588_MOESM2_ESM.pdf]

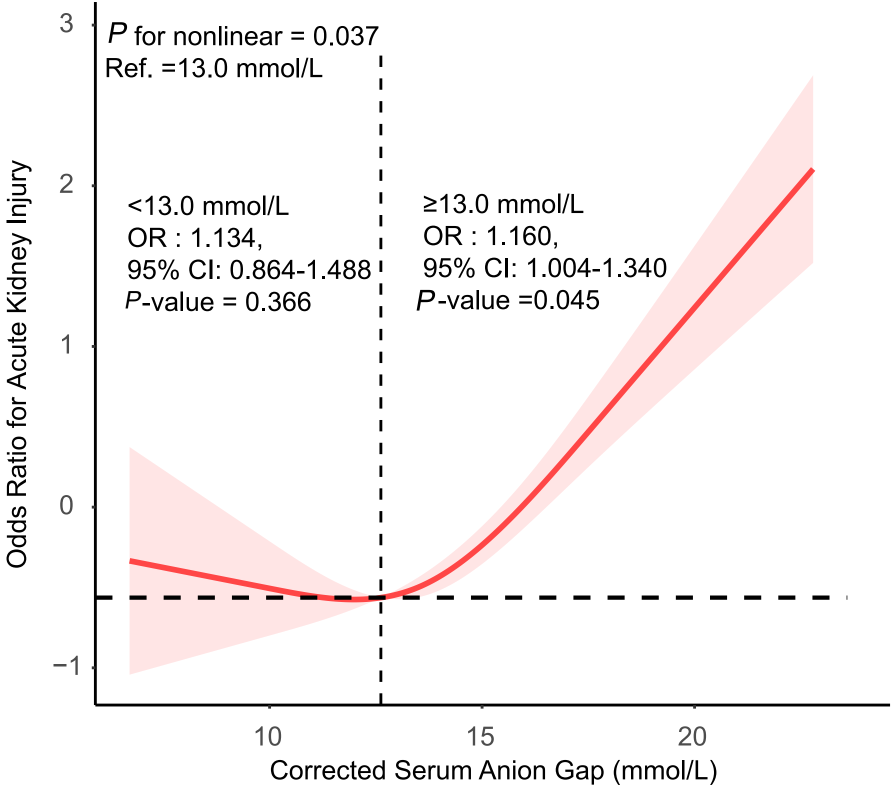

Supplementary Figure 1. Association between corrected serum anion gap and the risk of acute kidney injury after coronary artery bypass grafting
Abbreviation: OR, odd ratio; CI, confidence interval.
